# Restrictive influence of SAMHD1 on Hepatitis B Virus life cycle

**DOI:** 10.1038/srep26616

**Published:** 2016-05-27

**Authors:** Andreas F. R. Sommer, Lise Rivière, Bingqian Qu, Kerstin Schott, Maximilian Riess, Yi Ni, Caitlin Shepard, Esther Schnellbächer, Malin Finkernagel, Kiyoshi Himmelsbach, Karin Welzel, Nadja Kettern, Christian Donnerhak, Carsten Münk, Egbert Flory, Juliane Liese, Baek Kim, Stephan Urban, Renate König

**Affiliations:** 1 Host-Pathogen Interactions, Paul-Ehrlich-Institute, Langen, Germany; 2 Department of Infectious Diseases, Molecular Virology, University Hospital Heidelberg, Heidelberg, Germany; 3 Center for Drug Discovery, Department of Pediatrics, Emory Center for AIDS Research, Emory University, Children’s Healthcare of Atlanta, Atlanta, USA; 4 Division of Virology, Paul-Ehrlich-Institute, Langen, Germany; 5 Division of Medical Biotechnology, Paul-Ehrlich-Institute, Langen, Germany; 6 Clinic for Gastroenterology, Hepatology and Infectiology, Medical Faculty, Heinrich-Heine-University, Düsseldorf, Germany; 7 General and Visceral Surgery, Goethe-University, Frankfurt, Germany; 8 German Center for Infection Research (DZIF), Heidelberg, Germany; 9 Immunity and Pathogenesis Program, Sanford Burnham Prebys Medical Discovery Institute, La Jolla, CA, USA; 10 German Center for Infection Research (DZIF), Langen, Germany

## Abstract

Deoxynucleotide triphosphates (dNTPs) are essential for efficient hepatitis B virus (HBV) replication. Here, we investigated the influence of the restriction factor SAMHD1, a dNTP hydrolase (dNTPase) and RNase, on HBV replication. We demonstrated that silencing of SAMHD1 in hepatic cells increased HBV replication, while overexpression had the opposite effect. SAMHD1 significantly affected the levels of extracellular viral DNA as well as intracellular reverse transcription products, without affecting HBV RNAs or cccDNA. SAMHD1 mutations that interfere with the dNTPase activity (D137N) or in the catalytic center of the histidine-aspartate (HD) domain (D311A), and a phospho-mimetic mutation (T592E), abrogated the inhibitory activity. In contrast, a mutation diminishing the potential RNase but not dNTPase activity (Q548A) and a mutation disabling phosphorylation (T592A) did not affect antiviral activity. Moreover, HBV restriction by SAMHD1 was rescued by addition of deoxynucleosides. Although HBV infection did not directly affect protein level or phosphorylation of SAMHD1, the virus upregulated intracellular dATPs. Interestingly, SAMHD1 was dephosphorylated, thus in a potentially antiviral-active state, in primary human hepatocytes. Furthermore, SAMHD1 was upregulated by type I and II interferons in hepatic cells. These results suggest that SAMHD1 is a relevant restriction factor for HBV and restricts reverse transcription through its dNTPase activity.

Despite the existence of a vaccine, chronic hepatitis B virus (HBV) infection affects more than 350 million people worldwide and represents a leading cause of end-stage liver disease and hepatocellular carcinoma. HBV is a small, enveloped DNA virus with a 3.2 kbp partially double-stranded genome, which harbors four genes that encode seven proteins. Similar to retroviruses, its replication requires the reverse transcription of an RNA template (the pregenomic RNA, pgRNA) by the viral polymerase. However, unlike retroviruses, this event takes place during the late stages of the replication cycle, after encapsidation of the pgRNA into the capsid, and results in a partially double-stranded DNA (the relaxed circular DNA, rc-DNA). This rc-DNA represents the mature form of the HBV genome in the released viral particles. Upon infection of a new cell, the rc-DNA is imported into the nucleus and converted into a covalently closed circular DNA (cccDNA) that serves as a template for the transcription of all the viral RNAs^1^.

To fight against viral infections, cells have evolved intrinsic defense mechanisms. This first line of defense consists of constitutively expressed or interferon-induced restriction factors that inhibit viral replication. Factors, such as proteins of the APOBEC3 family, TRIM5α, tetherin, and SAMHD1 (sterile alpha motif and histidine-aspartate (HD)-domain-containing protein 1), as well as the respective viral counter-mechanisms, were identified during the last decade and representing promising targets for therapeutic strategies. Although most antiviral factors were initially discovered to suppress human immunodeficiency virus type-1 (HIV-1), further studies have shown that they possess a broader antiviral activity against various DNA or RNA viruses, including HBV^2^. For example, APOBEC and tetherin proteins have been demonstrated to inhibit HBV replication^3^,^4^,^5^,^6^,^7^,^8^,^9^,^10^.

SAMHD1 is a protein with a sterile α-motif and an HD domain that harbors the active site of the protein. It was recently identified as a restriction factor that inhibits HIV-1 in resting cells and has been shown to be degraded by the accessory factor Vpx, which is encoded by HIV-2 and the closely related simian immunodeficiency virus (SIV) strains, through a proteasome-dependent mechanism^11^,^12^,^13^. SAMHD1 is a GTP/dGTP-activated deoxynucleotide triphosphohydrolase (dNTPase) that is involved in the regulation of the cellular level of deoxynucleotide triphosphates (dNTPs)^14^,^15^. This activity requires homo-tetramerization of the protein^16^,^17^,^18^,^19^. The inhibitory mechanism against HIV-1 was first linked to the dNTPase activity of the protein, which lowers the intracellular dNTP concentration to below the level needed for viral reverse transcription^20^. Furthermore, it has been suggested that SAMHD1 or an associated protein could restrict HIV-1 through an RNase activity^16^,^21^. However, the relative contribution of these two activities in viral restriction is still a matter of debate^22^,^23^ and recent reports challenge the notion of an additional nuclease activity^24^,^25^. The antiviral activity of SAMHD1 is regulated through phosphorylation at threonine 592. In cycling cells, SAMHD1 is phosphorylated by cyclin-dependent kinase 1 or 2 (CDK1, CDK2), rendering SAMHD1 unable to inhibit retroviruses^26^,^27^,^28^. Dephosphorylation at residue T592 has been demonstrated to regulate the resistance to HIV-1 infection in non-cycling cells, such as myeloid cells or resting T cells^26^,^27^,^29^. However, whether the phosphorylation of SAMHD1 at residue T592 influences the enzymatic function of the protein remains controversial. On the one hand, it was suggested that this residue does not influence the dNTPase function^27^,^28^, whereas other studies propose that dNTPase-competent SAMHD1 homo-tetramers are destabilized through phosphorylation at T592^19^,^30^,^31^. Furthermore, phosphorylation at T592 was shown to negatively regulate the proposed RNase activity^16^. SAMHD1 is an interferon-induced factor. Its upregulation by interferon α (IFNα) was first shown in primary human monocytes^13^ and in various cell lines^32^,^33^,^34^, suggesting that its antiviral activity might not be limited to HIV-1 and could be involved in a broader antiviral defense. In support of this idea, in addition to HIV-1, SAMHD1 has been shown to restrict not only other types of retroviruses, such as SIV, feline immunodeficiency virus and equine infectious anemia virus^18^,^35^,^36^, but also DNA viruses, such as vaccinia virus and herpes simplex virus^37^,^38^, as well as LINE-1 and Alu/SVA retrotransposons^39^.

In a recent study, it was suggested that SAMHD1 can also restrict HBV^34^. This study demonstrated that overexpression of SAMHD1 reduces the production of HBV antigens, as well as viral DNA from a transfected HBV construct. Since previous studies have shown that the level of intracellular dNTPs affects HBV replication^40^,^41^,^42^, it stands to reason that SAMHD1 dNTPase activity would account for this restriction. However, Chen *et al*. suggested that the dNTPase activity of SAMHD1 might not be exclusively involved in the restriction since a catalytically inactive mutant of SAMHD1 was also able to inhibit HBV^34^. Nonetheless, the precise step of HBV replication affected by SAMHD1 and the mechanism involved remains unclear, as does the role of endogenous SAMHD1 in the context of HBV infection.

We investigated the antiviral effect of endogenous SAMHD1 on the complete HBV replication cycle using cell models stably transduced with short hairpin RNAs (shRNAs) targeting SAMHD1. We used both, an hepatic cell line containing an integrated HBV genome, HepG2.2.15^43^, and an infection model based on HepG2-NTCP cells^44^, which overexpresses the recently identified HBV receptor, sodium-taurocholate cotransporting polypeptide (NTCP)^44^,^45^. We show that depletion of SAMHD1 increased HBV replication in both cell lines, while overexpression had the opposite effect. We demonstrated that SAMHD1 affects both the level of HBV DNA in cell supernatants and the levels of intracellular HBV DNA, without affecting the levels of cccDNA or of any of the viral RNA subsets, suggesting that SAMHD1 targets reverse transcription but not the early stages of viral replication. Moreover, mutations that interfere with oligomerization and dNTPase activity (D137N) or the catalytic center of the HD domain (D311A)^16^,^21^, as well as phospho-mimetic mutation of T592 (T592E)^27^, abrogated restriction. In addition, SAMHD1 antiviral activity on HBV is counteracted by addition of exogenous deoxynucleosides (dNs). Together, these data demonstrate that SAMHD1 is a restriction factor for HBV that most likely affects reverse transcription through its dNTPase activity. Interestingly, we show that HBV upregulates the level of intracellular dATPs in infected cells without affecting the expression level or phosphorylation status of SAMHD1. SAMHD1 is upregulated by type I and II interferons, is expressed and in a de-phosphorylated potentially antiviral-active state in primary human hepatocytes, suggesting that SAMHD1 contributes to the antiviral effect of interferon *in vivo*.

## Results

### SAMHD1 restricts HBV replication in HepG2.2.15 cells

**Figure 1:**
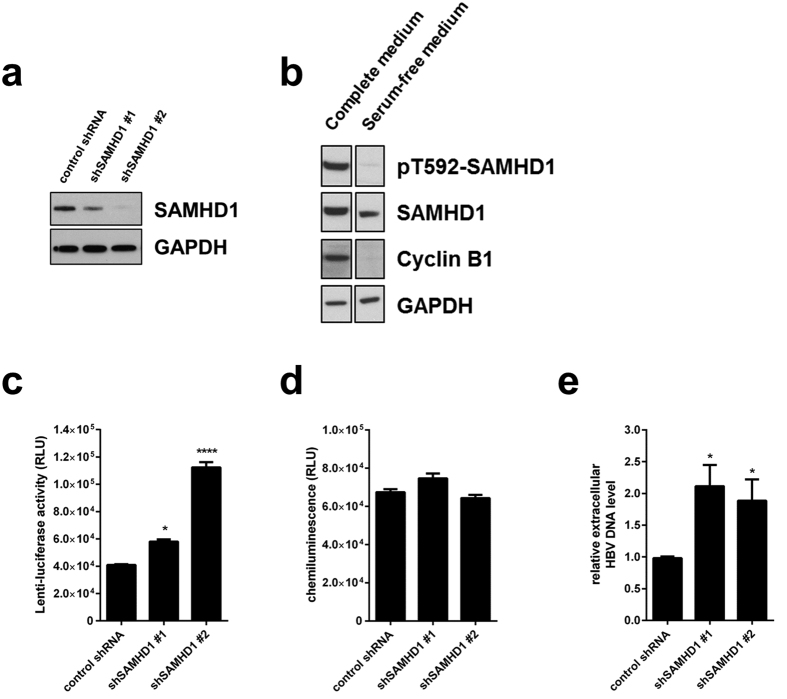
Silencing of SAMHD1 increases HBV replication in resting HepG2.2.15 cells. (**a**) SAMHD1 or actin protein levels were detected in cell lysates from SAMHD1 knockdown or control HepG2.2.15 cells by Western blotting. (**b**) Phosphorylation on residue T592 of SAMHD1 and cyclin B1 expression were detected by Western blotting of lysates from control shRNA HepG2.2.15 cells cultured for 3 days in complete versus serum-free medium. (**c-e**) HepG2.2.15 cells stably expressing two different SAMHD1 shRNAs or a control shRNA were cultured in serum-free medium for 3 days. (**c**) Luciferase activity (relative luciferase units, RLU) was detected in cells 24 hours post infection with a single-round HIV-1-based lentiviral vector that encoded luciferase. (**d**) Equal cell growth and viability between different shRNA cell lines was assessed using ATPLite. (**e**) The amount of HBV DNA in the supernatant was determined by qPCR and normalized to the control shRNA. In (**c**,**d**), the mean ± standard error mean (SEM) of three biological replicates of one representative experiment is depicted. In (**e**), the fold-changes to negative control were calculated for each individual experiment based on the median of three biological replicates, each measured in three technical replicates. The mean ± SEM of the fold-changes of four independent experiments is depicted. Statistical significance was determined using a one-way ANOVA with multiple comparisons according to Dunnett (*p < 0.05; ****p < 0.00005).

As a DNA virus, HBV depends on the availability of dNTPs in certain stages of its replication: in the early phase for completion of the incoming rc-DNA into cccDNA and in the late phase for the maturation of newly infectious particles during reverse transcription. First, we investigated the influence of SAMHD1 on the late HBV replication stages. For this purpose, we used HepG2.2.15 cells, which are stably transfected with an HBV genome, express all viral RNAs and proteins, produce viral genomes, and secrete viral particles^43^,^46^. Endogenous SAMHD1 was depleted in HepG2.2.15 cells by lentiviral transduction of two different shRNAs specifically targeting SAMHD1. Knockdown of SAMHD1 was confirmed by Western blotting (Fig. 1a). Since SAMHD1 has been shown to restrict viruses, such as HIV-1, only in non-cycling cells due to dephosphorylation at T592^26^,^27^, we arrested the cells through serum starvation. Western blot analysis of cyclin B1 confirmed the cell cycle arrest (Fig. 1b). Moreover, SAMHD1 was phosphorylated at T592 in cycling cells, but not in arrested cells (Fig. 1b). Under these conditions, silencing of SAMHD1 by two independent shRNAs (compared to a control shRNA) led to a substantial increase in transduction of an HIV-1 lentiviral vector encoding luciferase (Fig. 1c), without affecting cell growth and viability, compared to control cells, as measured by ATPlite (Fig. 1d), indicating that SAMHD1 inhibits the HIV-based vector in this cell system. To assess the role of SAMHD1 in the late HBV replication steps, we quantified the HBV DNA in the supernatant of SAMHD1 knockdown cells compared to control HepG2.2.15 cells cultured under serum-starvation conditions. The supernatant of SAMHD1-silenced cells contained significantly higher amounts of HBV DNA (Fig. 1e), demonstrating that a lack of SAMHD1 facilitates the production or release of DNA-containing HBV particles.

**Figure 2:**
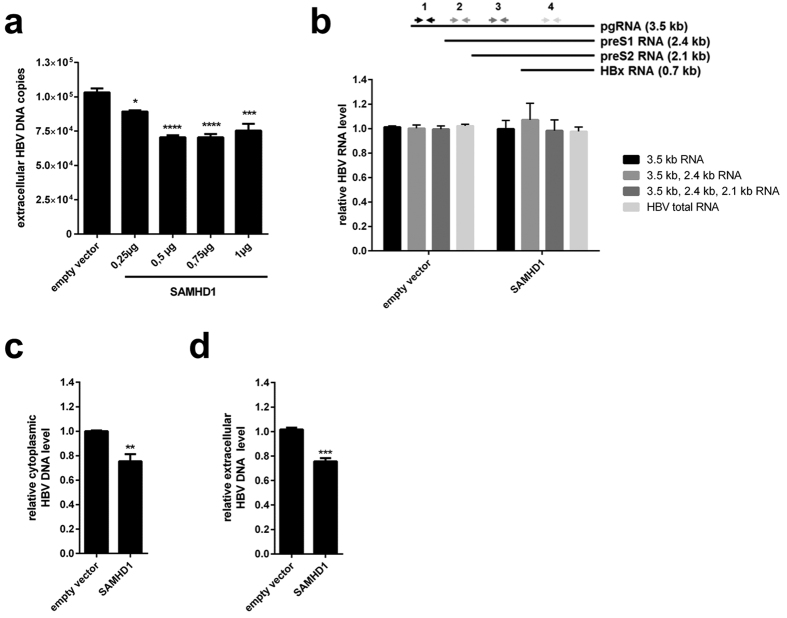
Overexpression of SAMHD1 reduces extracellular and intracellular levels of HBV DNA in HepG2.2.15 cells without affecting viral RNA. HepG2.2.15 cells were transfected with (**a**) increasing amounts or (**b**–**d**) 1 μg of a SAMHD1-coding plasmid. The total amount of plasmid DNA was adjusted to 1 μg by addition of empty vector. Twenty-four hours after transfection, the cells were washed with phosphate-buffered saline (PBS) and further cultured in serum-free medium without supplements. Forty-eight hours later, HBV DNA from the supernatant (**a**,**d**) and intracellular HBV DNA from cytoplasmic fractions (**c**) were quantified by qPCR. (**b**) The levels of intracellular HBV RNAs were determined by RT-qPCR using primer sets that amplify the indicated HBV RNAs (3.5 kb; 3.5 kb and 2.4 kb; 3.5 kb, 2.4 kb and 2.1 kb, or all four HBV RNAs). (**a**) The mean ± SEM of the technical triplicates is shown. Statistical significance was determined using a one-way ANOVA with multiple comparisons according to Dunnett (*p < 0.05; ***p < 0.0005; ****p < 0.00005). (**b**–**d**) For each independent experiment, fold-changes were calculated based on the median of three technical replicates divided by the median of three technical replicates of two negative controls. The mean ± SEM of the fold-changes of four independent experiments is represented. Statistical significance was determined using an unpaired t-test (**p < 0.005; ***p < 0.0005).

Next, we evaluated whether overexpression of SAMHD1 may have the opposite effect on HBV replication. Therefore, we transfected cDNA of SAMHD1 into HepG2.2.15 cells and assessed the levels of HBV DNA in the supernatant. Overexpression of SAMHD1 in HepG2.2.15 cells reduced the amount of HBV DNA-containing particles in the supernatant in a dose-dependent manner (Fig. 2a). Since HepG2.2.15 cells contain an integrated HBV genome, this result suggests that SAMHD1 affects a late stage of the HBV life cycle, between viral transcription and particle release. We then investigated the possible influence of SAMHD1 on viral transcription or RNA stability by analyzing intracellular HBV RNA from HepG2.2.15 cells overexpressing SAMHD1 (Fig. 2b). Specific primers were used to differentiate between HBV pgRNA and RNA subsets including successively preS1, preS2 and HBx RNA (Fig. 2b, upper panel). Interestingly, neither the HBV RNA levels of the 3.5 kb transcript (pgRNA) nor any of the RNA levels including smaller transcripts were significantly altered (Fig. 2b, lower panel) by SAMHD1 overexpression in comparison to control. This suggests that transcription of viral RNA from four different promotors and also the RNA stability is not affected by SAMHD1. Next, we used cytoplasmic fractions of cells overexpressing SAMHD1 to study the impact of SAMHD1 on newly transcribed HBV DNA levels (Fig. 2c). In comparison to control cells, the cytoplasmic HBV DNA levels (Fig. 2c) and concomitantly the release of HBV DNA (Fig. 2d) was significantly inhibited by SAMHD1, suggesting an effect of SAMHD1 on the product of reverse transcription. Thus, we conclude that SAMHD1 is a restriction factor for HBV that reduces the levels of viral DNA without affecting viral RNA levels, possibly by inhibiting the reverse transcription step.

### SAMHD1 restriction of HBV requires dNTPase activity and is sensitive to T592 phosphorylation

**Figure 3:**
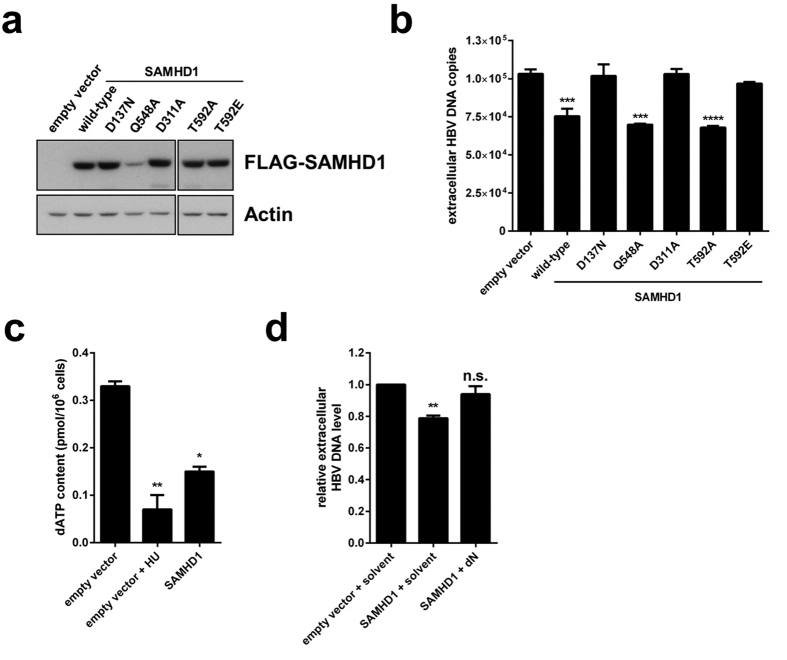
Restriction of HBV by SAMHD1 is dependent upon dNTPase activity, is inhibited by phosphorylation of T592 and is rescued by addition of deoxynucleosides. (**a**,**b**) HepG2.2.15 cells were transfected with 1 μg of plasmids coding for wild-type or mutant FLAG-tagged SAMHD1 or with 1 μg of empty vector. Twenty-four hours after transfection, the cells were washed and cultured in serum-free medium without supplements for 48 hours. (**a**) Detection of overexpressed FLAG-SAMHD1 by Western blotting. Detection of actin was used as a loading control. (**b**) HBV DNA from the supernatant was quantified by qPCR. The mean ± SEM of technical triplicates from one representative experiment out of three independent experiments is represented. (**c**) HepG2 cells were transfected with 1 μg of plasmid coding for wild-type FLAG-tagged SAMHD1 or empty vector. Twenty-four hours after transfection, the cells were washed and cultured in serum-free medium without supplements to arrest the cell cycle. When indicated, 10 mM of hydroxyurea (HU) were added at the time of medium change. Quantification of dATP levels 3 days post medium change were determined by single-nucleotide incorporation assay. The mean ± SEM of technical duplicates is represented. (**d**) HepG2.2.15 cells were transfected with 1 μg of plasmid coding for wild-type FLAG-tagged SAMHD1 or with an empty vector. Twenty-four hours after transfection, the cells were washed and cultured in serum-free medium without supplements. When indicated, deoxynucleosides (dNs, 2 mM each) or solvent were added at the time of medium change. HBV DNA from the supernatant was quantified by qPCR 3 days post medium change. For each independent experiment, fold-changes to empty vector were calculated based on the median of three technical replicates. The mean ± SEM of the fold-changes of three independent experiments is represented. (**b**,**d**) Statistical significance was determined using a one-way ANOVA with multiple comparisons according to Dunnett (**p < 0.005; ***p < 0.0005; ****p < 0.00005; n.s.: non-significant).

In order to assess the influence of the enzymatic function of SAMHD1 on the restriction of HBV replication, we transfected HepG2.2.15 cells with FLAG-tagged wild-type SAMHD1 or tagged mutants that are deficient for the dNTPase (D137N), the potential RNase activity (Q548A), or both enzymatic functions (D311A)^16^,^21^. In addition, cells were transfected with SAMHD1 variants, wherein the residue T592, which is important for HIV restriction, was replaced by a phospho-mimetic (T592E) or a non-phosphorylatable residue (T592A). Protein levels were monitored by Western blotting (Fig. 3a) and HBV replication was assessed by qPCR of the HBV DNA in the cellular supernatant (Fig. 3b). Interestingly, the catalytically inactive mutant D311A fully abrogated the restriction of HBV compared to wild-type SAMHD1, clearly demonstrating that the inhibitory effect of SAMHD1 is based on its enzymatic function. Despite the lower expression level of the mutant Q548A in comparison to equally high levels of the other mutants and wild-type SAMHD1 (Fig. 3a), SAMHD1 Q548A, which lacks the potential RNase activity, was able to restrict HBV as efficiently as the wild-type protein. In contrast, the D137N mutant, which lacks the dNTPase activity, as well as the D311A mutant, which is impaired in both, the dNTPase and putative RNase activity, fully abrogated the restriction of HBV compared to wild-type SAMHD1. Consistent with the dNTPase activity of SAMHD1, the intracellular dATP pools were significantly reduced more than 50% by SAMHD1 in resting hepatic cells (Fig. 3c). As a control, we treated the cells with hydroxyurea (HU) that inhibits the enzyme ribonucleotide reductase (RNR) converting rNDP into dNDP resulting in decreased dNTP pools, as measured by significant low dATP levels^47^. Next, we reasoned that if virus replication is limited due to the dNTPase function of SAMHD1 resulting in reduced dNTP availability, providing an exogenous source of dNTPs would relieve the restriction to HBV infection. Therefore, we added to the medium pyrimidine and purine deoxynucleosides (dNs) as dNTP precursors to increase the dNTP pool^20^. We found that dN treatment counteracted the inhibitory activity of SAMHD1 as compared to solvent-treated control cells (Fig. 3d). These results demonstrate that the SAMHD1 inhibitory effect is based on its dNTPase activity and not on the proposed RNase activity.

We next sought to define the contribution of T592 phosphorylation on HBV replication. The SAMHD1 non-phosphorylatable mutant T592A inhibited HBV as efficiently as the wild-type protein. In contrast, the phospho-mimetic mutant T592E abrogated the effect (Fig. 3b).

In summary, these results indicate that restriction of HBV by SAMHD1 is dependent on its dNTPase activity and is abrogated by phosphorylation on residue T592.

### SAMHD1 restricts HBV replication in the HepG2-NTCP infection model by inhibiting the reverse transcription step

**Figure 4:**
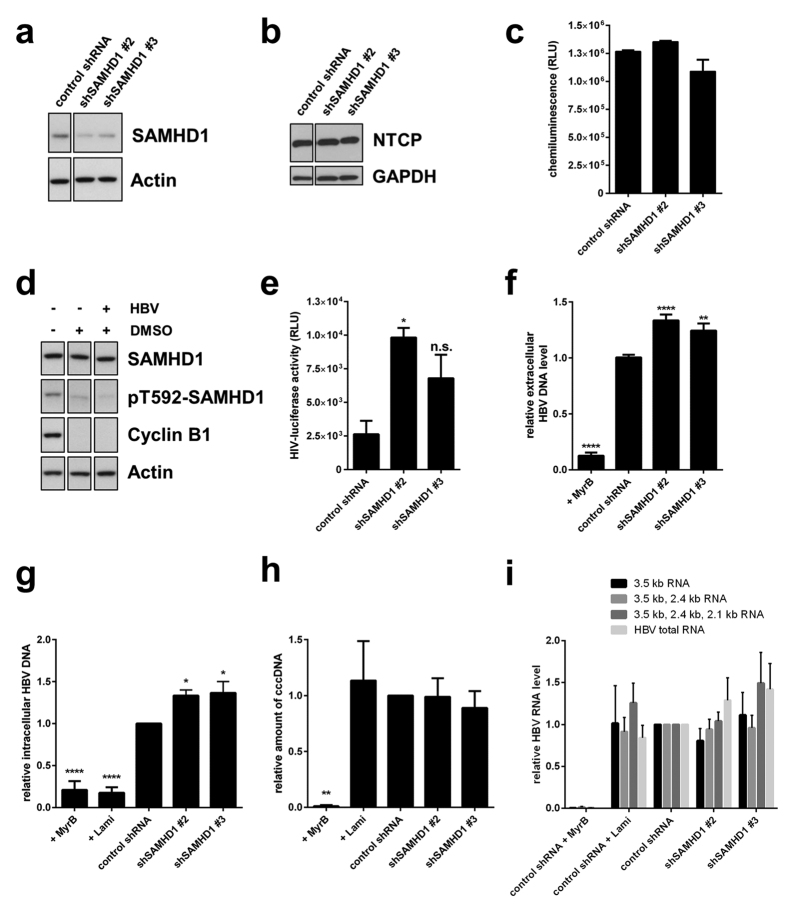
SAMHD1 restricts HBV replication in infected HepG2-NTCP cells. Detection of (**a**) SAMHD1 or (**b**) NTCP in SAMHD1 knockdown or control HepG2-NTCP cells by Western blotting. Actin or GAPDH were used as loading controls. (**c**) Equal cell growth and viability was assessed was assessed using ATPLite. Depicted are mean values (±SEM) of three biological replicates of one representative experiment out of three. (**d**) Control shRNA cells were cultured for 10 days in complete medium supplemented (+) or not (−) with 2.5% DMSO. When indicated cells were infected with HBV 48 hours after start of DMSO treatment. Phosphorylation at residue T592 in SAMHD1, as well as expression of total SAMHD1, cyclin B1 and actin, were detected by Western blotting. (**e**) Cells were cultured for 10 days in 2.5% DMSO-containing medium and infected with a single-round luciferase HIV-1 virus. Luciferase activity was detected 24 hours post infection. Depicted are mean values (±SEM) of three biological replicates of one representative experiment out of three. (**f-i**) Cells were infected with HBV inoculum, and where indicated, the entry inhibitor MyrcludexB (MyrB, 200 nM), or the reverse transcription inhibitor lamivudine (Lami, 0,5 μM) were added. Cells were cultured in medium containing 2.5% DMSO. Ten days post infection, HBV DNA from the supernatant (**f**), intracellular total HBV DNA (**g**) and cccDNA (**h**) were quantified by qPCR. (**i**) The intracellular HBV RNAs levels were determined 10 days post infection by RT-qPCR using primer sets that amplify the indicated HBV RNAs (3.5 kb; 3.5 kb and 2.4 kb; 3.5 kb, 2.4 kb and 2.1 kb, or all four HBV RNAs). (**f**) For each individual experiment, fold-changes to the untreated control shRNA were calculated based on the median of three biological replicates, each measured in three technical replicates. The mean ± SEM of the fold-changes of three independent experiments is depicted. (**g**–**i**) For each independent experiment, fold-changes to untreated control shRNA were calculated based on the median of three technical replicates. The mean ± SEM of the fold-changes of at least 3 independent experiments is represented. (**f**–**i**) Statistical significance was determined using a one-way ANOVA with multiple comparisons according to Dunnett (*p < 0.05, **p < 0.005; ****p < 0.00005).

To investigate the influence of endogenous SAMHD1 on the full HBV replication cycle, we used HepG2-NTCP cells that stably express the Na^+^ -taurocholate cotransporting polypeptide (NTCP), the receptor for HBV, and that support effective viral infection^44^,^45^. HepG2-NTCP cells were transduced with specific SAMHD1 shRNA or scrambled shRNA lentiviral vectors. Depletion of SAMHD1 was confirmed by Western blotting (Fig. 4a). Knockdown of SAMHD1 did not alter the expression level of NTCP (Fig. 4b), cell viability or growth (Fig. 4c). The cell cycle was arrested by adding 2.5% dimethylsulfoxide (DMSO) into the cell medium 48 hours prior to infection. This method was chosen over the serum starvation used earlier because it allows for better cellular adherence and viability during the time of infection. Cell cycle arrest was confirmed by the reduced protein levels of cyclin B1 (Fig. 4d). Furthermore, arrested cells showed reduced phosphorylation at SAMHD1 residue T592 (Fig. 4d). To confirm that SAMHD1 possessed antiviral activity under these culture conditions, cells were infected with an HIV-1-based single-round infection luciferase reporter virus, and luciferase activity was measured 24 hours post infection (Fig. 4e). Considerably enhanced luciferase activity (shSAMHD1 #2: 3,7-fold; shSMAHD1 #3: 2,6-fold) could be detected in SAMHD1 knockdown cells, indicating that the HIV-1 reporter virus was restricted by SAMHD1 and that the protein retained its antiviral activity in the control cells under these culture conditions. Cell cycle-arrested control or SAMHD1 knockdown HepG2-NTCP cells were infected with HBV particles and viral replication was assessed 10 days post infection by quantification of HBV DNA in the supernatant (Fig. 4f). To ensure that the DNA signal arises from viral replication and not from the remaining inoculum, the HBV entry inhibitor Myrcludex B (MyrB)^48^ was included in a control. Significantly lower amount of viral DNA could be amplified from supernatants of MyrB-treated cells compared to non-treated cells, thus excluding the possibility of contamination from the inoculum. Interestingly, silencing of SAMHD1 significantly increased the level of released viral DNA compared to the control (Fig. 4f), suggesting that SAMHD1 mitigates the production or release of DNA-containing HBV particles in a *bona fide* infection model. Since the full HBV life cycle is reflected in this model, the dNTPase function of SAMHD1 could possibly affect an early and a late step where dNTPs are needed for the synthesis of HBV DNA: the completion of incoming rcDNA into cccDNA, which serves as a template for viral transcription, and the DNA product of reverse transcription, respectively. The HBV infected HepG2-NTCP control or SAMHD1 silenced cells were monitored for both, the levels of intracellular HBV DNA reflecting the product of reverse transcription and the levels of cccDNA (Fig. 4g,h). In controls, MyrB or the reverse transcriptase inhibitor lamivudine were included. Significantly lower amount of both, intracellular HBV DNA and cccDNA, could be amplified in MyrB-treated cells compared to non-treated cells, ensuring that the measured DNA signal does not arise from the inoculum. As expected, lamivudine inhibited significantly only the intracellular DNA (Fig. 4g), whereas the levels of cccDNA were not affected (Fig. 4h) ensuring the specificity of the chosen primers and assay system. Interestingly, in the HepG2-NTCP infection model, SAMHD1 knockdown increases the level of intracellular viral DNA compared to the control (Fig. 4g), without significantly affecting the level of cccDNA (Fig. 4h). We reasoned that an equal level of cccDNA template would, in consequence, also lead to equal viral RNA transcript levels. HBV total RNA, the pgRNA as well as pgRNA together with smaller transcripts are not considerably affected by silencing of SAMHD1 (Fig. 4i) indirectly confirming the unaffected cccDNA levels. Moreover, these findings validate the data from Figs 2b and 3b and confirm that transcription of viral RNAs or RNA stability is not affected by SAMHD1. In summary, these data suggest that SAMHD1 acts late in the life cycle inhibiting the reverse transcription stage. Overall, these data indicate that SAMHD1 restricts HBV replication in a *bona fide* infection model, thus indicating that SAMHD1 is a relevant restriction factor in the HBV life cycle.

### HBV does not alter the protein levels or de-phosphorylation status at T592 of SAMHD1 in primary hepatocytes

**Figure 5:**
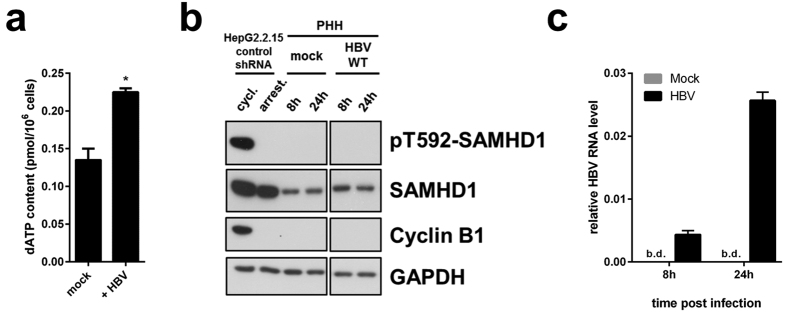
dATP levels are affected by HBV infection. SAMHD1 is de-phosphorylated at T592 in primary human hepatocytes, and is not affected by HBV infection. (**a**) HepG2-NTCP- control-shRNA cells were infected or not with HBV in presence of 2.5%DMSO and dATP content was measured 4 days post infection. The mean ± SEM of technical duplicates is represented. (**b**, **c**) Primary human hepatocytes (PHH) from one donnor were infected or not with HBV for 8 or 24 hours. (**b**) SAMHD1 phosphorylation on T592, total SAMHD1, cyclin B1 and GAPDH were detected by Western blotting. Cycling or serum starved HepG2.2.15-control-shRNA cells were used respectively as positive and negative controls for pT592-SAMHD1 and cyclin B1. A representative experiment out of two technical replicates is represented. (**c**) Levels of intracellular HBV RNAs from infected PHH used in (**b**) were determined by RT-qPCR using HBV primer set 3 (see Fig. 2b and Table 1; amplified HBV RNA species: 3.5 kb, 2.4 kb and 2.1 kb RNAs). B.d.: below detection. The mean ± SEM of technical triplicates is represented.

Viruses have evolved sophisticated means to evade or directly counteract restriction factors. Thus, we investigated if HBV would counteract the action of SAMHD1 by directly affecting SAMHD1 expression/phosphorylation or by indirect means, for instance, affecting the dNTP synthesis pathway. It has been shown that HBV can promote deoxynucleotide synthesis by activating the RNR-R2 pathway in quiescent HepG2.2.15 cells compared to quiescent HepG2 cells^42^, however these data were not yet confirmed in an infection model. Thus, we measured the concentration of dATP in quiescent HepG2-NTCP control-shRNA cells infected for 4 days with HBV. Interestingly, HBV significantly increases the level of dATP compared to non-infected cells (Fig. 5a) in concordance with Cohen *et al*.^42^. Intriguingly, in this cell system, HBV does not alter the expression level or the phosphorylation at T592 of SAMHD1 compared to non-infected cells (Fig. 4d).

Next, we investigated if SAMHD1 would directly regulate the expression or the phosphorylation status of SAMHD1 in primary hepatocytes (PHH). We infected PHH with HBV for 8 h or 24 h and compared the SAMHD1 status to uninfected PHH by Western blotting (Fig. 5b). As a control, we included cycling and arrested HepG2.2.15 control-shRNA cells. Cyclin B1 expression and phosphorylation at T592 of SAMHD1 could only be detected in cycling HepG2.2.15 cells, validating that the PHH are indeed in a quiescent state. Moreover, we did not observe any phosphorylation signal on T592 in PHH consistent with previous reports demonstrating that SAMHD1 is dephosphorylated in resting cells^26^,^31^. HBV infection in PHH was monitored by measuring viral RNA transcripts (Fig. 5c). Interestingly, HBV infection did not significantly affect the expression level of SAMHD1 or its phosphorylation on T592 in PHH at both time points (Fig. 5b). Since we demonstrated in Fig. 3b that SAMHD1 is only active against HBV when dephosphorylated at T592, this result suggests that SAMHD1 is active in PHH and thus a relevant restriction factor against HBV *in vivo,* but not directly antagonized by the virus.

### SAMHD1 expression is induced by type I and type II interferons

**Figure 6:**
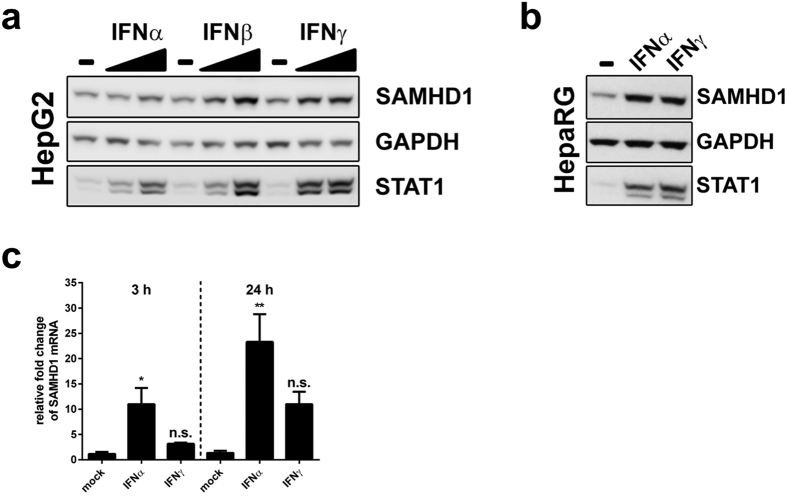
Interferon induces SAMHD1 expression in hepatic cell lines and in primary human hepatocytes. (**a**) HepG2 were cultured with 20 or 2000 U/mL, while (**b**) HepaRG cells were cultured with 2000 U/mL of interferon α2a (IFNα2a), interferon β (IFNβ) or interferon γ (IFNγ) for 20 hours. Cells were collected, lysed and probed for SAMHD1, STAT1 and GAPDH by Western blotting. (**c**) Primary human hepatocytes (PHH) from one donor were treated with 500 U/ml of IFNα2a or IFNγ for 3 or 24 hours. The relative level of SAMHD1 mRNA was determined by RT-qPCR. Fold-changes to untreated PHHs at the respective time points were calculated based on the median of three technical replicates. The mean ± SEM of the fold-changes of three technical replicates is represented. Statistical significance was determined using a one-way ANOVA with multiple comparisons according to Dunnett (*p < 0.05, **p < 0.005, n.s.: non-significant).

We next tested whether SAMHD1 is an interferon-induced protein in hepatic cells since interferon responsiveness is one of the hallmarks of an antiviral factor^49^. HBV infection has been shown to be controlled by interferon and upregulation of interferon-stimulated genes (ISGs)^3^,^6^,^50^,^51^,^52^,^53^. We reasoned that SAMHD1 might be a contributing factor in the interferon-induced innate response towards HBV. Therefore, we first determined the protein expression levels of SAMHD1 upon treatment with type I (IFNα, IFNβ) or type II interferons (IFNγ) in the hepatocellular carcinoma cell line HepG2, as well as in HepaRG, a human hepatic progenitor cell line that retains many characteristics of primary human hepatocytes (Fig. 6a,b). We could demonstrate a dose-dependent upregulation of SAMHD1 expression in HepG2 cells with both type I and II interferons (Fig. 6a). IFNα and IFNγ also boosted the expression of SAMHD1 protein in HepaRG cells (Fig. 6b). Moreover, we determined the mRNA expression level of SAMHD1 in PHH upon IFNα and IFNγ treatment (Fig. 6c). SAMHD1 mRNA levels were increased at 3 hour and 24 hour by 9.4-fold and 17.1-fold, respectively, after IFNα treatment and by 2.7 and 8.1-fold, respectively, upon IFNγ addition, thus confirming that SAMHD1 is an ISG in hepatic cells.

## Discussion

For the first time, the role of endogenous SAMHD1 in HBV replication has been addressed. Moreover, a *bona fide* infection model, HepG2-NTCP, was used to elucidate the impact of SAMHD1 on HBV replication. A previous study reported that overexpression of SAMHD1 could inhibit HBV replication in hepatic cell lines transfected with HBV genome^34^. Here, we confirm these results and extend the findings to demonstrate that endogenous SAMHD1 affects cytosolic HBV DNA production and release, but does not affect viral RNA subsets. Using several lines of evidence, we further explore the mechanism of action to reveal that the dNTPase activity of SAMHD1 is responsible for the inhibitory effect. Moreover, we suggest that SAMHD1 is a relevant restriction factor in primary hepatocytes *in vivo*.

We demonstrate that knockdown of endogenous SAMHD1 in HBV-producing HepG2.2.15 cells enhances the release of HBV DNA into the supernatant, while SAMHD1 overexpression has the opposite effect (Figs 1e and 2a). SAMHD1 overexpression affects the level of intracellular cytoplasmic DNA (Fig. 2c), demonstrating that SAMHD1 does not inhibit viral particles release. Interestingly, we observed no significant effect of SAMHD1 on the level of any HBV RNA subset in HepG2.2.15 cells that overexpressed SAMHD1 (Fig. 2b), suggesting that SAMHD1 restricts a later stage of HBV replication after RNA transcription, possibly the reverse transcription. Furthermore, for the first time, we used an infection model based on SAMHD1 silenced HepG2-NTCP cells to prove the relevance of endogenous SAMHD1 restriction on HBV infection. We found that knockdown of SAMHD1 increased both HBV DNA levels in supernatant and intracellular replication intermediates in infected cells (Fig. 4f,g). On the contrary, SAMHD1 had no significant effect on the levels of cccDNA or on any HBV RNA subset (Fig. 4h,i), confirming that SAMHD1 antiviral activity targets a late stage of HBV replication, but does not significantly affect early stages such as completion of rcDNA into cccDNA or transcription. These results show for the first time the antiviral activity of endogenous SAMHD1 on HBV replication of hepatocytes.

In contrast to the findings of Chen *at al*.^34^, in our experiments, mutation of the active center of the HD domain (D311A) abrogated the inhibitory effect of SAMHD1 (Fig. 3b) suggesting the requirement of an enzymatic function for SAMHD1 in order to inhibit HBV. The mutant used by Chen *et al*. (HD/AA) was, in our hands, considerably less expressed than the wild-type SAMHD1 and aggregated in the nucleus, therefore we excluded it from our analysis (data not shown). The mutation Q548A—which is described as having a robust dNTPase activity, while its RNAse activity is impeded^16^—limited viral production to the same level as the wild-type SAMHD1. In contrast, we found that the mutation of D137—an amino acid residing in the allosteric site and involved in dGTP binding, which is necessary for oligomerization of SAMHD1—rendered SAMHD1 inactive against HBV. This suggests that the dNTPase activity of SAMHD1, which requires oligomerization^17^, is the major contributor to HBV restriction. Additionally, this mutant was described to retain the putative RNase activity. Its lack of antiviral activity against HBV, which is consistent with the findings that HBV RNA is not affected by SAMHD1 (Figs 2b and 4i), rules out the involvement of viral RNA degradation in the restriction of HBV by SAMHD1. Furthermore, addition of exogenous dNs to HepG2.2.15 cells can overcome the restriction of HBV by SAMHD1 (Fig. 3d), most likely by rescuing the depletion of intracellular dNTPs by SAMHD1 in hepatic cells (Fig. 3c). These results confirm the involvement of the dNTPase activity of SAMHD1 in HBV restriction. In summary, our results suggest that SAMHD1 acts on the reverse transcription step of HBV replication.

Moreover, we found that phosphorylation at T592, which is only present in cycling cells, is linked to an antiviral-inactive state of SAMHD1. While SAMHD1 T592A inhibited the production of viral DNA, the phospho-mimetic mutant T592E had no effect, indicating that the antiviral activity is regulated through cell cycle-dependent phosphorylation similar to that in HIV-1^26^,^27^. Additionally, we demonstrated that SAMHD1 is expressed and de-phosphorylated at T592, and thus potentially active in primary quiescent hepatocytes (Fig. 5b) indicating that SAMHD1 is a relevant factor to be taken into account *in vivo*. It is proposed that phosphorylation of T592, which negatively regulates SAMHD1’s restrictive capacity, could also influence its dNTPase activity, potentially through destabilization of the catalytically active homotetratmers^19^,^24^,^27^,^28^,^30^,^31^. However, it was reported that a phosphomimetic substitution of T592 is still able to reduce the intracellular dNTP levels in human myeloid cells while having no effect on viral restriction^27^. Though the mechanistic implication of phosphorylation at T592 is still a matter of debate, HBV replication is sensitive to T592 phosphorylation.

While our data clearly demonstrate that SAMHD1 is a restriction factor for HBV, the virus is able to replicate and spread in resting cells in culture and in the liver despite the presence of restriction-competent SAMHD1. Therefore, we can propose that its restriction mechanism would not absolutely protect a cell from becoming infected, but would rather slow down viral spread. Interestingly we observed an increase in dATP level in HBV infected Hep2-NTCP cells (Fig. 5a) which is in concordance with a previous report^42^ suggesting that HBV could partially overcome the effect of SAMHD1. Based on our results in Fig. 4, this mechanism may not be sufficient to fully antagonize SAMHD1, since we clearly see an effect upon silencing of SAMHD1 on HBV DNA levels. We hypothesize that the rise of dATP levels by HBV is not due to direct action on SAMHD1, but rather indirectly through manipulation of the *de novo* dNTP synthesis pathway. We show that HBV infection does not directly antagonize SAMHD1 through controlling its expression or phosphorylation status as shown in HepG2-NTCP cells (Fig. 4d) or in PHH (Fig. 5b), though we cannot exclude other mechanism used by HBV to directly block SAMHD1 function. It has been described that the accessory protein HBx of HBV is capable of manipulating the cellular dNTP pool through upregulation of the R2 subunit of the ribonuclease reductase^42^,^54^. Thus, this promotes *de novo* synthesis of dNTPs, counteracting the dNTP-regulatory function of SAMHD1. Since HBx is believed to not be incorporated into viral particles^55^, viral replication kinetics would be limited by SAMHD1 until there was sufficient expression of HBx. Moreover, besides a time-dependent component of the balance between SAMHD1 inhibitory effect and antagonism by HBV, the virus is probably very sensitive to fluctuations in dNTP levels despite its ability to upregulate the dNTP pool. The HBV polymerase displays only 62% activity at dNTP concentration of 0,4 μM^56^ which is the concentration measured in resting adult liver cells^42^. Additional influence of SAMHD1 might tip the balance to inhibition of HBV in hepatocytes *in vivo*.

Our data suggest HBV as a relevant restriction factor in primary hepatocytes (Fig. 5b). We show that SAMHD1 expression is upregulated by type I and type II IFNs in hepatic cell lines as well as in PHH (Fig. 6). Moreover, several studies have substantiated the upregulation of SAMHD1 by IFNα in primary human liver cells^57^,^58^ placing SAMHD1 as a novel ISG in hepatocytes. Innate mechanism that have been proposed to be intact in hepatic cells^57^ could sense HBV resulting in upregulation of SAMHD1 and subsequent inhibition^59^,^60^,^61^.

We report here, for the first time, that SAMHD1 restricts HBV in an infection model in hepatic cells. Our data suggest that restriction of HBV by SAMHD1 occurs by depletion of cellular dNTPs, rather than by degradation of viral RNA. Nevertheless, we cannot formally exclude an additional mechanism based on the interaction of SAMHD1 with viral RNA or inhibition of RNA encapsidation. SAMHD1 has been reported to show alternative RNA-binding activity^62^,^63^ besides its nuclease function^16^,^64^, which may mediate HIV-1 restriction^16^. Moreover, our data suggest the following model: *in vivo*, SAMHD1 may be active in hepatocytes, but its antiviral activity could be weakened by upregulation of dNTPs by the virus, through an independent pathway, most likely via RNR-R2 activation^42^,^54^. Under these conditions, SAMHD1 may slow down but not completely stop viral replication. However, under conditions leading to SAMHD1 upregulation, such as interferon treatment or innate immunity, SAMHD1 antiviral activity would become prominent. Together with other recently described interferon-induced restriction factors, such as members of the APOBEC family or tetherin, SAMHD1 would then contribute to a strong inhibition of viral replication. Recognizing SAMHD1 as a relevant restriction factor in HBV infection will lead to novel insights into HBV replication and potentially new antiviral treatments.

## Methods

### Plasmids

Point mutations (D137N, Q548A, D311A, T592A, T592E) in SAMHD1-coding plasmid pcDNA3.1-N-FLAG-SAMHD1^13^ were introduced by site-directed mutagenesis using complementary primer containing respective nucleotide exchanges. Mutagenesis PCR was performed with *Pfu*Ultra (Agilent Technologies) or KOD Hot Start (Merck Milipore) DNA Polymerase. Non-mutated template DNA was subsequently removed by *Dpn*I (New England Biolabs) treatment of the PCR product. Mutations were verified by DNA sequencing.

The pJO19 HBV coding plasmid was linearized and used as a standard in quantification of HBV DNA copies in quantitative PCR (qPCR).

### Cell culture and generation of knockdown cell lines

HepG2-NTCP cells are derived from HepG2 cells and have been stably transfected with HBV receptor, the sodium-taurocholate cotransporting polypeptide (*NTCP*)^44^. HepG2.2.15 cells are derived from HepG2 cells and have been stably transfected with HBV genome^43^,^46^.

HepG2.2.15, HepG2, HepG2-NTCP and derived cell lines were cultivated in Dulbecco’s modified eagle medium (DMEM) containing 10% fetal calf serum (FCS), 2 mM L-Glutamine and 1% Penicillin/Streptomycin (“complete DMEM”) at 37 °C. Cells were seeded in poly-L-Lysine (PLL) coated plates. When indicated, cell cycle arrest was induced by withdrawal of supplements or addition of 2.5% dimethyl sulfoxide (DMSO) to culture medium. HepaRG cells were cultivated in Williams E medium containing 10% fetal calf serum (FCS), 5 μg/ml Insulin, 50 μM hydrocortisone hemisuccinate and 1% Penicillin/Streptomycin (complete Williams E medium) at 37 °C. For treatment with interferons, the cells were cultured as indicated in medium containing the indicated concentration of human interferon α2a, β (PBL interferon source) or γ (Peprotech) respectively.

SAMHD1 knockdown cell lines were generated by transduction of HepG2.2.15 or HepG2-NTCP cells with lentiviral vectors encoding SAMHD1 specific shRNA (shSAMHD1 #1: TRCN0000343807 5′-CCCTGAAGAAGATATTTGCTT-3′/shSAMHD1 #2: TRCN0000343808 5′-GCCATCATCTTGGAATCCAAA-3′/shSAMHD1 #3: TRCN0000343809 5′-GCCAGTGCTAAACCCAAAGTA-3′) or nonspecific control shRNA (Non-target or shc204) (TRCN library, Broad institute, distributed by SIGMA Aldrich) and subsequent selection with 3 μg/ml puromycin. In case of HepG2-NTCP, the *puromycin* selection gene in pLKO vectors was exchanged by *hygromycin*, since HepG2-NTCP cells already carry a puromycin resistence gene. Transduced HepG2-NTCP cells were selected using 2 mg/ml of hygromycin. Primary human hepatocytes (PHH) were isolated from liver specimens resected from patients undergoing partial hepatectomy. Approval from the local and national ethics committees (Ethic committee from the Goethe University of Frankfurt, agreement number 343/13) and informed consent from patients were obtained. PHH were isolated with a two-step perfusion method and cultured as described previously^65^). PHH were maintained in William’s E medium (Gibco-Invitrogen) with 5% FCS, 7 × 10–5 M hydrocortisone hemisuccinate, 5 μg/ml insulin and 2% DMSO (Sigma-Cell Culture reagent).

### Cell Transfection

HepG2.2.15 cells were seeded on PLL coated plates in complete DMEM and transfected with 1 μg plasmid DNA using X-tremeGene HP (ROCHE) 24 hours later. When indicated, total amount of plasmid DNA was complemented to 1 μg with empty vector (pcDNA3.1). The following day, transfected cells were washed three times in PBS to remove viral particles from cell surface and further on cultured in DMEM without supplements in order to arrest cell cycle. Samples were analyzed 72 hours later.

### Drugs

For deoxynucleosides (dNs) treatment, a mix of four dNs (2′-deoxyguanosine, Thymidin, 2′-deoxyadenosine, 2′-deoxycytidine, Sigma-Aldrich) dissolved in RPMI-HCl pH 4,1 were supplemented to the cells to a final concentration of 2 mM each. The same volume of RPMI-HCl pH 4,1 was added to the control cells.

When indicated, hydroxyurea (Sigma-Aldrich) was added to the cells to a final concentration of 10 mM,

### Cell viability

Cell viability was observed using ATPLite (Perkin Elmer), which detects intracellular ATP, according to manufacturer’s instructions. Chemiluminescence of ATPLite, measured in relative light units (RLU), was detected using a PherastarFS plate reader (BMG).

### Reporter virus and shRNA lentiviral vector production and transduction

Production of HIV-1 based single round luciferase reporter virus (HIV-luc) was performed by transfection of HEK293T cells with an HIV-1 based luciferase reporter virus defective in *env* (pNL43lucE-R+^66^), and pseudotyped with VSV-G coding expression plasmid^67^ in a ratio of 2:1 using Lipofectamine2000 according to the manufacturer’s instructions (Invitrogen/Life Technologies). A third generation lentiviral single round luciferase reporter vector (lenti-luc) was produced as previously described (CSII-luc^68^). Viral particles were harvested 48 and 72 hours post transfection, filtered with 0.45 μm pore size sterile filter and stored at −80 °C. For transduction, reporter viruses were added to cells and spinoculated at 290 g at 30 °C for 90 minutes. Luciferase activity was measured 24 hours after transduction using BriteLite reagent according to manufacturer’s protocol (PerkinElmer). Chemiluminescence, measured in relative light units (RLU), was detected using a PherastarFS plate reader (BMG).

### Hepatitis B Virus preparation and infection

Preparation of Hepatitis B Virus inoculum from HepAD38 cells^69^ was performed as previously described^44^. For infection, HepG2-NTCP SAMHD1 knockdown or control shRNA cell lines were seeded into PLL coated plates in complete DMEM supplemented with 2.5% DMSO for 48 hours. HepG2-NTCP-shRNA or PHH cultivated as described above were inoculated with HBV in presence of 4% Polyethylene glycol 8000 (PEG). When indicated, HBV entry inhibitor MyrcludexB^48^ [200 nM] or reverse transcriptase inhibitor lamivudine [0,5 μM]^6^ were added at the time of infection. Approximately 16 hours after infection, cells were washed three times with PBS and fresh medium was added. The medium was changed every 72 hours and lamivudine was renewed.

### Preparation of viral DNA and quantitative PCR analysis

The sequence and position of all primers used for qPCR and RT-qPCR is indicated in Table 1.

For quantification of HBV copy numbers in supernatant of HepG2.2.15 cells or HBV infected HepG2-NTCP cells, viral DNA was isolated using the High Pure Viral Nucleic Acid Kit (ROCHE diagnostics) according to manufacturer’s protocol.

Purified DNA was used for real time PCR with Maxima SYBR Green qPCR Master Mix (Thermo Scientific) and HBV DNA specific primers (HBV RC F; HBV RC R, Table 1). Absolute copy numbers were calculated referring to a standard based on the linearized HBV coding plasmid pJO19.

For analysis of intracellular total HBV DNA and cccDNA from infected HepG2-NTCP cells, DNA from total cell lysates was prepared using the NucleoSpin Tissue Kit (Macherey-Nagel 740952.250) according to manufacturer’s protocol with minor modifications. Briefly, cells were trypsinized and re-suspended in provided T1 buffer and incubated with proteinase K and B3 buffer at 70 °C for 1 hour and following steps were stick to the protocol. For cytosolic HBV DNA in HepG2.2.15 cells, cells were treated with fractionation buffer (10 mM HEPES, 1.5 mM MgCl_2_, 10 mM KCl) containing 1% NP-40. NP-40 soluble fraction was transferred and mixed with proteinase K and B3 buffer and cytosolic DNA was obtained using the same kit. For cccDNA quantification, DNA samples were digested with T5 exonuclease (New England Biolabs. M0363). Products were subjected for qPCR with cccDNA specific primers spanning the gap region). This protocol was shown to specifically allow the detection of cccDNA and to avoid contamination by rcDNA^70^ (Qu *et al*., manuscript in preparation). β-globin expression levels from undigested samples were used for normalization (β-globin-F; β-globin-R). For HBV total DNA, qPCR reactions were performed on undigested samples in SYBR green supermix (Bio-rad 172-5121) using HBV specific primers HBV-total-DNA-F and HBV-total-DNA-R (95 °C for 10 minutes, followed up by 95 °C for 10 seconds and 60 °C for 30 seconds for 40 cycles). A plasmid carrying two copies of full-length HBV genome was used for standard curve^71^.

### RNA extraction and reverse transcription quantitative PCR analysis

For RT-qPCR analysis, total RNAs were extracted from cell pellets using NucleoSpin® RNA Plus Kit (Macherey Nagel). Reverse Transcription Quantitative Real-Time PCR (RT-qPCR) was performed using QuantiTect SYBR Green RT-PCR Kit (Qiagen) using specific primers. For analysis of HBV RNAs, primers sets that specifically amplify the following HBV RNA subsets were used: 3.5 kb RNA only (HBV RNA 1 F; HBV RNA 1 R); 3.5 kb and 2.4 kb RNAs (HBV RNA 2 F; HBV RNA 2 R); 3.5 kb, 2.4 kb and 2.1 kb RNAs (HBV RNA 3 F; HBV RNA 3 R); or all four HBV RNAs (“total HBV RNAs”, HBV RNA 4 F; HBV RNA 4 R, Table 1 and Fig. 2b upper panel). SAMHD1 mRNAs were detected using the primers hSAMHD1_F and hSAMHD1_R (Table 1). As a reference gene RPL13A (RPL13A_F; RPL13A_R, Table 1) was used. For each sample, the relative amount of RNA was calculated using the formula 2^−ΔCt^ with ΔCt = (Ct HBV − Ct RPL13A). When indicated, relative fold change to the control condition was calculated using the formula 2^−ΔΔCt^ with ΔΔCt = ΔCt_sample_ − ΔCt_control_ = (Ct HBV_sample_ − Ct RPL13A_sample_) − (Ct HBV_control_ − Ct RPL13A_control_).

### Immunoblot analysis

Cell samples were lysed using a TritonX-100 based Lysis Buffer (100 mM NaCl, 10 mM EDTA, 20 mM TRIS pH7.5, 1% TritonX-100, 1% Sodiumdesoxycholate). For detection of NTCP, samples were additionally treated with PNGaseF (New England Biolabs) according to manufacturer’s protocol. Proteins were separated by SDS-PAGE and transferred to a Nitrocellulose membrane. Specific proteins were detected using anti-SAMHD1 (Proteintech), anti-pT592-SAMHD1 (from Prof. Dr. Oliver Keppler, Max von Pettenkofer-Insitute, Ludwig Maximilians-University, Munich, Germany, or from Thermo Fisher Scientific), anti-FLAG (M2, Sigma-Aldrich), anti-NTCP (Sigma-Aldrich), anti-CyclinB1 (Cell-Signaling), anti-STAT1 (Santa Cruz), anti-Actin (Sigma-Aldrich), anti-GAPDH (Cell-signaling) antibodies and respective horseradish peroxidase (HRP) conjugated secondary antibodies (GE Healthcare).

### Intracellular dNTP measurement

For dNTP analysis and quantification, cells were harvested and lysed in ice cold 65% methanol, and vigorously vortexed for 2 min. Extracts were incubated at 95 °C for 3 min. Supernatants were collected and dried in a speed vacuum. Samples were processed for the single nucleotide incorporation assay as described^72^.

### Statistical analysis

Each group was compared to the respective negative control. Statistical significance of differences was analyzed with an unpaired t-test or a one-way ANOVA with multiple comparisons according to Dunnett. A *p* value of (p) < 0.05 was considered statistically significant.

## Additional Information

**How to cite this article**: Sommer, A. F. R. *et al*. Restrictive influence of SAMHD1 on Hepatitis B Virus life cycle. *Sci. Rep.* **6**, 26616; doi: 10.1038/srep26616 (2016).

## Figures and Tables

**Table 1:** Primer list.

**Name**	**Sequence**	**For HBV, position relative to the EcoRI site**	**Usage**
HBV RNA 1 F	TGTCAACACTAATATGGGCCTAA	2164–2186	HBV 3.5 kb RNA (RT-qPCR)
HBV RNA 1 R	AGGGGCATTTGGTGGTCTAT	2295–2314	
HBV RNA 2 F	ACAAGGTAGGAGCTGGAGCA	2981–3000	HBV 3.5 kb and 2.4 kb RNAs (RT-qPCR)
HBV RNA 2 R	GTAGGCTGCCTTCCTGACTG	3112–3131	
HBV RNA 3 F	GCTTTCACTTTCTCGCCAAC	1087–1106	HBV 3.5 kb, 2.4 kb and 2.1 kb RNAs (RT-qPCR)
HBV RNA 3 R	GAGTTCCGCAGTATGGATCG	1262–1281	
HBV RNA 4 F	GTGCACTTCGCTTCACCTCT	1579–1598	total HBV RNAs (RT-qPCR)
HBV RNA 4 R	AGCTTGGAGGCTTGAACAGT	1859–1878	
hSAMHD1_F	TCACAGGCGCATTACTGCC		SAMHD1 mRNAs (RT-qPCR)
hSAMHD1_R	GGATTTGAACCAATCGCTGGA		
HBV RC F	CAC TCT ATG GAA GGC GGG TA	2753–2772	HBV extracellular DNA (qPCR)
HBV RC R	TGC TCC AGC TCC TAC CTT GT	2981–3000	
HBV-total-DNA-F	GTTGCCCGTTTGTCCTCTAATTC	465–487	intracellular or cytoplasmic HBV DNA (qPCR)
HBV-total-DNA-R	GGAGGGATACATAGAGGTTCCTTGA	540–564	
β-globin-F	CAGGTACGGCTGTCATCACTTAGA		normalisation of qPCR
β-globin-R	CATGGTGTCTGTTTGAGGTTGCTA		
RPL13A_F	CCT GGA GGA GAA GAG GAA AGA GA		normalisation of RT-qPCR
RPL13A_R	TTG AGG ACC TCT GTG TAT TTG TCA A		
